# Decreased Rivaroxaban Levels in a Patient with Cerebral Vein Thrombosis Receiving Phenytoin

**DOI:** 10.1155/2017/4760612

**Published:** 2017-08-10

**Authors:** Ana F. Becerra, Tomas Amuchastegui, Aldo H. Tabares

**Affiliations:** ^1^Vascular Medicine and Thrombosis Service, Hospital Privado Universitario de Cordoba, Cordoba, Argentina; ^2^Instituto Universitario de Ciencias Biomedicas de Cordoba (IUCBC), Cordoba, Argentina; ^3^Internal Medicine Service, Hospital Privado Universitario de Cordoba, Cordoba, Argentina

## Abstract

Combined use of antiepileptic drugs and anticoagulants is common. We describe the first case documenting laboratory interaction between rivaroxaban and phenytoin. A 48-year-old woman was admitted to our hospital due to cerebral venous thrombosis, bilateral pulmonary embolism, and deep vein thrombosis. She came from a small town with difficult access to warfarin monitoring. She was receiving phenytoin 100 mg three times daily (t.i.d.) and started enoxaparin 60 mg twice daily (b.i.d.). An abdominal mass was diagnosed and removed by laparoscopy (gastrointestinal stromal tumor). On day 5, she was switched to rivaroxaban 15 mg b.i.d. First peak anti-Factor Xa was 70 ng/ml (reference value: 100–300 ng/ml). She was discharged on rivaroxaban 15 mg b.i.d. and phenytoin 100 mg t.i.d. A week later, anti-Xa levels were 90 ng/ml. Due to concerns about thrombosis progression, she was switched to dabigatran. During follow-up, she remained asymptomatic and thrombin time >180 s was measured several times along 3 months as surrogate for dabigatran activity. Phenytoin is a combined CYP3A4 and P-glycoprotein inducer, which might reduce rivaroxaban levels. Dabigatran is substrate of P-glycoprotein, meaning potential malabsorption. Despite unavailability of plasmatic dabigatran essays, our patient improved her symptoms without further symptomatic thromboembolism. Facing these interactions, either monitoring serum levels of anticoagulants or other therapeutic options should be considered.

## 1. Introduction

The introduction of Direct Oral Anticoagulants (DOACs), such as the thrombin-inhibitor dabigatran and the Factor Xa-inhibitors rivaroxaban, apixaban, and edoxaban, has provided a safe and effective alternative to vitamin-K antagonists (VKA) for the prevention of systemic embolism in patients with nonvalvular atrial fibrillation and both prevention and treatment of venous thromboembolism. Despite these many advances, there still remain some gaps regarding the pharmacology and clinical relevance of the pharmacokinetics of these drugs.

Rivaroxaban is a Factor Xa-inhibitor agent that is a substrate for the hepatic cytochrome P450 3A4 (CYP3A4); it is mainly eliminated through renal permeability glycoprotein (P-gp) efflux transporter protein system. Dabigatran, instead, is not a CYP3A4 but a P-gp substrate [1, 2].

A history of stroke accounts for about 30–40% of acquired epilepsy in the elderly, placing the concomitant use of antiepileptic agents and DOACs in a rather frequent situation [2].

The pharmacokinetics and pharmacodynamics of antiepileptic drugs are heterogeneous and complex and much of these aspects have been well studied both in in vitro and in animal models, but unfortunately little human research has yet been conducted. The induction of CYP3A4 activity by phenytoin has been clearly documented in humans, but P-gp induction has only been demonstrated in animals (Phase I studies) [2, 3]. We describe the first case of documented interaction between rivaroxaban and phenytoin.

## 2. Case Report

A 48-year-old woman was admitted to the hospital due to cortical cerebral venous thrombosis with small areas of hemorrhage. She had been previously admitted to a medical center in her hometown in suspicion of stroke, where she was initially stabilized, and then referred to our hospital for further evaluation. Her first symptoms consisted of generalized seizures for which she was started on phenytoin 100 mg t.i.d. On admission to our institution, she complained of left leg pain and dyspnea and was subsequently diagnosed with bilateral pulmonary thromboembolism and left iliac and femoral vein thrombosis.

She had no family history of thrombosis nor cancer, or toxic habits. She referred to changes in her bowel movements but had not been further studied. She denied weight loss, fever, headaches, or dyspnea. As a single thrombotic risk factor, she had been taking contraceptives for menorrhagia for several months prior to admission and received no other drugs.

At presentation, the patient was oriented and hemodynamically stable and needed no oxygen supplementation. Physical examination revealed slight weakness of her left lower limb and tenderness to palpation of the left lower abdominal quadrant. Her body mass index was 27 (weight 60.3 kg). Her creatinine clearance calculated by MDRD (Modification of Diet in Renal Disease) formula was 118 ml/min and platelet count was 231,000 mm^3^. Liver enzymes and coagulation assays were reported within normal ranges (prothrombin time, Kaolin activated Partial Thromboplastin Time (KPTT), and thrombin time (TT)). She was admitted to general wards and was started on anticoagulant therapy with low molecular weight heparin (LMWH). Phenytoin was not discontinued due to previous seizures. A thrombophilia panel ruled out Protein S deficiency, Protein C deficiency, antiphospholipid syndrome, and activated Protein C resistance. A CT scan of the abdomen showed a left lower quadrant mass, so she underwent laparoscopic resection of the mass on the third hospital day. Histopathology of the mass revealed a low-grade gastrointestinal stromal tumor of the gut. Twelve hours after surgery, she resumed enoxaparin and at fifth hospital day, she was started on rivaroxaban 15 mg b.i.d. because the patient came from a small city with rather difficult access to VKA monitorization.

After the second dose of rivaroxaban, a peak anti-Factor Xa—3 hours after taking the medication—showed levels of 70 ng/ml (reference value: 100–300 ng/ml). While waiting for rivaroxaban to reach a steady state, the patient was discharged under supervision of home-care service, both on rivaroxaban 15 mg b.i.d. and on phenytoin 100 mg t.i.d. A week later, she visited the Vascular Medicine Service and at that time, a chromogenic anti-Xa level, measured 3 hours after taking rivaroxaban, was 90 ng/ml. Upon further questioning, the patient denied noncompliance with the medications. Even though the patient had remained asymptomatic, she was switched to dabigatran because of concerns about recurrent thromboembolism associated with low rivaroxaban plasmatic levels. During follow-up visits, she remained asymptomatic, showing good clinical response, and a TT >180 seconds, 4 hours after taking dabigatran, was measured in three separate opportunities along 2 months as an indirect index of dabigatran activity.

## 3. Discussion

To our knowledge, only three cases of interactions between rivaroxaban and antiepileptic drugs have been reported. The first one is a 53-year-old man who underwent an orthopedic surgery and suffered from pulmonary embolism, whose symptoms started one day after he was switched from prophylactic dalteparin to rivaroxaban 10 mg one a day (q.d.). The patient also used carbamazepine 600 mg b.i.d. for epilepsy; the authors hypothesized that carbamazepine as a CYP3A4 inducer had led to a decrease in the levels of rivaroxaban resulting in pulmonary embolism [4].

The second case is a 68-year-old man with epilepsy under oxcarbazepine therapy, who had been newly diagnosed with atrial fibrillation and started on rivaroxaban 20 mg q.d. After 6 weeks of treatment, transesophageal echocardiography showed a thrombus in the left atrial appendage and anticoagulation was switched to VKA aiming at an international normalized ratio target of 2-3. A repeated transesophageal echocardiography six weeks later demonstrated resolution of the thrombus. In this case, the authors pointed out that oxcarbazepine, a strong inducer of CYP3A4, might have led to a decreased anticoagulant activity of rivaroxaban resulting in intracardiac thrombus formation [5].

The third case is an 88-year-old poly morbid and poly medicated woman with AF and a nonconvulsive epileptic state, who was started on rivaroxaban irrespective of renal failure. After 3 days of treatment, rivaroxaban was discontinued due to concerns about bleeding risk. Coagulation tests 28 h after rivaroxaban intake showed INR 2.26, PT 35%, aPTT 38.3 s, and anti-Factor Xa-activity 2.00 U/ml. Explanations for the prolonged anticoagulant activity of rivaroxaban include renal failure, low body mass index, advanced age, and potential drug interaction between rivaroxaban and mirtazapine, valproic acid, and amlodipine [6].

Furthermore, Altena et al. reported a fatal case of pulmonary embolism due to a suspected interaction between rivaroxaban and rifampicin. The authors were able to document a low peak level of rivaroxaban of 178 ng/ml from a stored blood sample. They concluded that low rivaroxaban plasma levels could be attributed to the simultaneous administration of a strong CYP3A4 inducer such as rifampicin [7]. However, target therapeutic ranges for each DOACs have not been clearly established nor validated with respect to clinical outcomes [8, 9].

There is controversial evidence about these drug-drug interactions. Regarding available data, phenytoin is a combined strong CYP3A4 and P-gp inducer, which might cause the reduction in rivaroxaban plasma levels. Interestingly, patients under phenytoin or other combined inducers of CYP and permeability glycoproteins were excluded from the ROCKET-AF study [10]. Thus far, little evidence about the reduction of rivaroxaban plasma levels associated with concomitant use of antiepileptic drugs in humans has been reported and, more importantly, no clear evidence about their clinical relevance has been documented.

With regard to dabigatran, it is known that neither dabigatran etexilate nor its hydrolyzed form interacts with CYP3A4. In contrast, dabigatran etexilate is a substrate of P-gp transporters whereas the hydrolyzed form of dabigatran is not. Thus, potential pharmacokinetic drug-drug interactions involving P-gp are restricted to absorption across the intestinal wall (where P-gp is present). To date, only one phase I study has been conducted with dabigatran etexilate and a P-gp inducing agent. In this study, after 7 days of pretreatment with rifampicin 600 mg/d, dabigatran plasma levels of healthy volunteers were reduced by 67%. Seven days after cessation of rifampicin, dabigatran plasma levels returned to those seen when dabigatran was administered alone [3, 11]. However, Wiggins et al. reported a case which showed that dabigatran plasmatic levels were reduced after concomitant administration of phenytoin; nonetheless this was a 45-year-old very ill man who was poly medicated and the interaction could not be clearly established due to the numerous drugs he was taking [12]. Even though we were not able to measure dabigatran plasmatic levels, our patient improved her symptoms without further episodes of venous thromboembolism.

We are aware of some limitations regarding our report. First of all, the woman did not have any thrombotic event while being under rivaroxaban, and therefore the clinical effect of the interaction could not be documented. However, due to the persistently low rivaroxaban levels we decided not to take the risk to expose our patient to an otherwise avoidable event. Second, we have used chromogenic anti-Xa to assess rivaroxaban levels with a calibration curve constructed by spiking citrated normal platelet-poor plasma with rivaroxaban (Xarelto™) stock solution of 10 *μ*g/ml. As suggested by Pathak et al. [13], a greater variability in the rivaroxaban concentration was observed in the middle or higher part of the titration curve; therefore we are confident that the values obtained by our anti-Xa assay truly represent a low value [14]. Unfortunately, data regarding the most effective concentration of rivaroxaban are yet limited [15]. Finally, although it would have been interesting to check rivaroxaban levels prior to the introduction of phenytoin, we could not accomplish this because the patient had already started phenytoin before her arrival to our hospital and her risk of recurrent seizures was deemed too high.

In conclusion, to our knowledge, this is the first report of the interaction between rivaroxaban and phenytoin based on patient's plasmatic concentrations of the drug. Clinicians should be aware of such potential interactions and consider either monitoring serum levels or switching to other therapeutic options. The interaction between antiepileptic drugs and DOACs requires further research.
